# Diabetic Foot Infection Presenting Systemic Inflammatory Response Syndrome: A Unique Disorder of Systemic Reaction from Infection of the Most Distal Body

**DOI:** 10.3390/jcm8101538

**Published:** 2019-09-25

**Authors:** Cheng-Wei Lin, Shih-Yuan Hung, Chung-Huei Huang, Jiun-Ting Yeh, Yu-Yao Huang

**Affiliations:** 1 Division of Endocrinology and Metabolism, Chang Gung Memorial Hospital at Linkou, Taoyuan 33305, Taiwan; mushiau@gmail.com (C.-W.L.); 9002053@gmail.com (S.-Y.H.); a22932@cgmh.org.tw (C.-H.H.); 2 Department of Plastic surgery, Chang Gung Memorial Hospital at Linkou, Taoyuan 33305, Taiwan; yates1965@gmail.com; 3 College of Medicine, Chang Gung University, Taoyuan 33305, Taiwan; 4 Department of Medical Nutrition Therapy, Chang Gung Memorial Hospital, Taoyuan 33305, Taiwan

**Keywords:** diabetic foot infection, systemic inflammatory responsive syndrome, lower-extremity amputation, prognostic factors

## Abstract

Diabetic foot infection (DFI) is a major complication of diabetic foot that lead to nontraumatic lower-extremity amputation (LEA). Such distal infection of the body having systemic inflammatory response syndrome (SIRS) is rarely reported. Consecutive patients treated for limb-threatening DFI in a major diabetic foot center in Taiwan were analyzed between the years 2014 to 2017. Clinical factors, laboratory data, perfusion, extent, depth, infection and sensation (PEDIS) wound score in 519 subjects with grade 3 DFI and 203 presenting SIRS (28.1%) were compared. Major LEA and in-hospital mortality were defined as poor prognosis. Patients presenting SIRS had poor prognosis compared with those with grade 3 DFI (14.3% versus 6.6% for major LEA and 6.4% versus 3.5% for in-hospital mortality). Age, wound size, and HbA1c were independent risk factors favoring SIRS presentation. Perfusion grade 3 (odds ratio 3.37, *p* = 0.044) and history of major adverse cardiac events (OR 2.41, *p* = 0.036) were the independent factors for poor prognosis in treating patients with DFI presenting SIRS. SIRS when presented in patients with DFI is not only limb- but life-threatening as well. Clinicians should be aware of the clinical factors that are prone to develop and those affecting the prognosis in treating patients with limb-threatening foot infections.

## 1. Introduction

Diabetic foot ulcers (DFU) are prone to be infected because of impaired immunity, skin and nail disorders, peripheral arterial disease, neuropathy, and foot anatomy in patients with diabetes [1]. Among the diabetic foot complications, diabetic foot infection (DFI) is the leading threatening problem for limb loss and sepsis [2,3,4], and is the most common cause of hospital admissions [5,6] and costly expenditure [7] in diabetic populations. Among the in-hospital DFU cases, 82% have been reported to have DFIs in Europe and 94% in Taiwan [8,9].

Systemic inflammatory response syndrome (SIRS) indicates the inflammation beyond the infected site that traverses throughout the entire body. It is defined when matching two or more of the four abnormal presentations for body temperature, pulse rate, respiratory rate, and leukocyte count [10]. The presentation of SIRS is defined as the most severe grade of infection of various sites [11,12] suggesting higher risks to shock or mortality [10,13]. The presentation of SIRS in DFI, however, has been less reported. Wukich et al. reported that patients presenting with SIRS had higher major lower-extremity amputations (LEAs) and longer hospital stay compared to grade three of DFI [14].

From the mechanistic view, the infection of distal foot to affect the systemic reaction might be due to differences from SIRS of other foci of the body, especially when it has been associated with peripheral arterial disease (PAD) or neuropathy [1,15]. This study aimed to further understand the factors that are prone to development of SIRS in patients with DFI and factors affecting its prognosis of treatment.

## 2. Materials and Methods

### 2.1. Subjects and DFI Treatments

Consecutive type 2 diabetic patients with DFI treated at the major diabetic foot center in Taiwan, the Chang Gung Memorial Hospital at Linkou (a 3,700-bed university hospital), were reviewed from 2014 to 2017. Two hundred and three patients presenting with SIRS and 519 subjects with moderate DFI according to the Infection Disease Society of American (IDSA) [12] and the International Working Group on the Diabetic Foot (IWGDF) [11] criteria were identified. The Institutional Review Board of Chang Gung Memorial Hospital approved this study (no. 201900075B0). All patients received comprehensive foot care by a multidisciplinary team [16,17,18,19]. The empiric antibiotics were prescribed promptly for these patients initially, and subsequently modified according to the results of cultures. Surgical interventions, endovascular treatments, or foot amputations were scheduled after the diabetic foot team reached a consensus.

### 2.2. DFI and Wound Scoring

The wound classification was recorded as PEDIS describing the perfusion, extent size, depth/tissue loss, infection, and sensation of the wounds [11]. Patients presenting with SIRS were defined by grade 4 DFI. The definition of SIRS was according to matching two or more of the four criteria including abnormal body temperature >38 °C or <36 °C; tachycardia with pulse > 90 beat per minute; abnormal respiratory rate with > 20 breaths per minute; and abnormal leukocyte count > 12000 or < 4000 /cu mm. Patients with grade 3 DFI (presenting with either erythema >2 cm around the wound or involving the structures deeper than skin and subcutaneous tissues, but no SIRS) were included for comparison.

Their perfusion status was categorized into three grades. No symptom or sign of PAD was defined as grade 1; any symptom or sign of PAD or any non-invasive assessment document but not reaching critical limb ischemia was defined as grade 2; and critical limb ischemia was defined as grade 3. The critical limb ischemia in this study was defined by presence of gangrene or ulcers with ankle pressure <70 mmHg [20], or monophasic wave form of arteries below-the-knee [17]. Adjunct angiography was performed for confirmation.

### 2.3. Data Collection

Demographic information was recorded from the patients’ first visit at admission. The medical records included the patient’s age, gender, diabetes duration, HbA1c level, and medical history such as hypertension, history of major adverse cardiac events (MACEs; including coronary artery disease and cerebrovascular accidents), and dialysis. Smokers were classified as currently smoking if they smoked at least one cigarette per day. Their estimated glomerular filtration rate (eGFR) was calculated using the Modification of Diet in Renal Disease Study equation: 175 × serum creatinine (exp[−1.154]) × age (exp[0.203]) × (0.742 if female). Renal status was categorized as follows: Normal or mild chronic kidney disease (eGFR ≥ 60 mL/min); moderate to severe chronic kidney disease (eGFR < 60 mL/min); and dialysis according to the National Kidney Disease Outcomes Quality Initiative (NKDOQI) guidelines.

### 2.4. Prognosis Analysis: LEAs and in-Hospital Mortality

Status at discharge was stratified into four groups: Limb-preserved, minor LEA (i.e., amputation performed including digital amputation or tarsal-metatarsal amputation, as long as it did not involve the ankle area), major LEA (i.e., amputation performed above the ankle joint), or in-hospital mortality. The major LEA and in-hospital mortality were defined as poor prognosis while subjects with limb preservation and minor LEA were used for comparison.

### 2.5. Statistics

Age, duration of diabetes, wound extent size, and HbA1c level were used as continuous variables, and gender, smoking status, comorbidities, and PEDIS score (except extent size) were used as categorical variables. Comparisons between patients with or without SIRS were performed using the Mann–Whiney test for continuous variables and Pearson’s chi-square test for categorical variables. Each factor odds ratio to the presence of SIRS was calculated via adjusted model of logistic regression. The same statistical method was used in comparing the two groups with different treatment outcome of the total DFI patients with SIRS. The significant risk factors in the univariate analysis found above were then entered into a multivariate logistic regression model to identify independent risk factors to adverse outcome among these patients. All statistical analyses were performed using the Statistical Package for the Social Sciences (SPSS for Windows, version 19.0, IBM Corp., Armonk, NY, USA) data analysis software.

## 3. Results

### 3.1. The Characteristics of Patients DFI Presenting SIRS

The comparison of clinical characteristics of subjects, feet, and treatments between patients with grade 3 DFI and those with SIRS is shown in the Table 1. Patients presenting with SIRS had median age of 60.3 years and diabetes duration of 12 years, which was significantly lower than those with grade 3 DFI (age 66.0 y/o and duration 15 years, *p* < 0.001, and *p* = 0.04, respectively). Male gender predominance was noted in both groups (61.6 and 61.5%, in SIRS and grade 3 DFI, respectively). The rates of associated comorbidities were high in patients of both groups. For example, the MACE history was 30.0% versus 36.0% and dialysis was 17.2 versus 19.3% for presenting SIRS versus grade 3 DFI, respectively. In the presentation of DFUs, patients with SIRS had larger wound size compared to those with grade 3 DFI (15.0 cm versus 9.0 cm, *p* < 0.001) while no differences were found in perfusion, depth, or sensation. Osteomyelitis was noted of 40.4% in SIRS and 38.9% in grade 3 DFI (*p* = 0.716). Of note, patients with SIRS had relatively shorter days before visit (*p* = 0.066), longer stay of in-hospital treatment (*p* < 0.001), and higher blood levels of HbA1c (9.6% versus 8.1%, *p* < 0.001).

### 3.2. Factors Prone to Have SIRS Presentation

**Figure 1:**
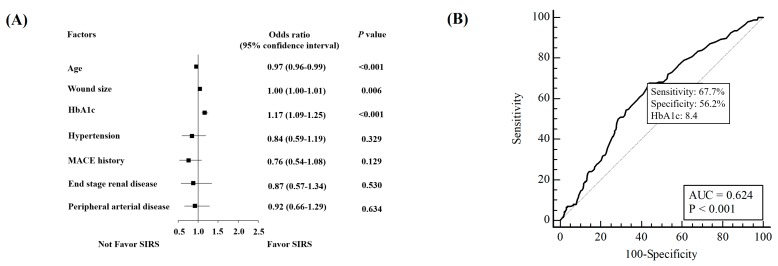
Factors associated with systemic inflammatory response syndrome (SIRS) presenting in DFI patients. (**A**) Forest plot of odds ratios under multivariate regression analysis, adjusted with age, wound size, HbA1c, hypertension, major adverse cardiac event (MACE) history, end-stage renal disease, and peripheral arterial disease. (**B**) Receiver operating characteristic (ROC) curve of HbA1c in predicting SIRS. According to Youden index, cutoff point in HbA1c: 8.4 had 67.7% sensitivity and 56.2% specificity; AUC, area under the curve.

When putting factors with statistical significance into the multivariate logistic regression model, the forest plot analysis of factors favored in presenting SIRS in patients with DFIs was demonstrated, as shown in Figure 1A. Age (odds ratio 0.97, 95% confidence interval 0.96–0.99, *p* < 0.001), wound size (OR 1.00, 95% CI 1.00–1.01, *p* = 0.006), and HbA1c (OR 1.17, 95% CI 1.09–1.25, *p* < 0.001) were the independent risk factors to predict SIRS. Analysis of HbA1c level at visiting via receiver operating characteristic (ROC) curve showed that the cutoff point was 8.4% with 67.7% sensitivity and 56.2% specificity in predicting SIRS (Figure 1B).

### 3.3. Prognosis and Prognostic Factors Analysis in Patients Presenting SIRS

**Figure 2:**
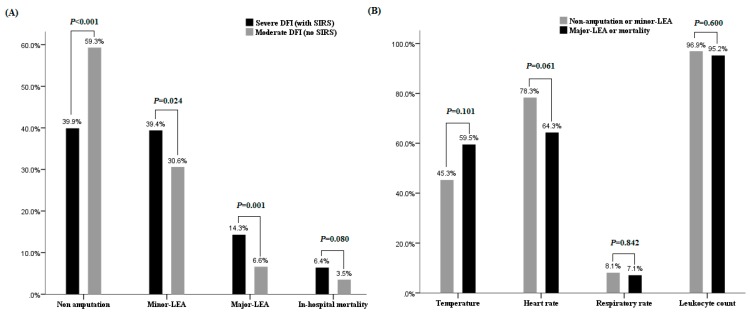
Treatment outcome results and associated factors for severe DFI patients with SIRS. (**A**) The prognosis of patients with grade 3 DFI and those with SIRS. Patients with SIRS had higher major lower-extremity amputation (LEA) (*p* = 0.001) and relatively higher in-hospital mortality (*p* = 0.08); (**B**) The presentation of individual component of SIRS did not affect the prognosis.

Among the 203 patients with SIRS, 42 patients had poor prognosis. Twenty-nine patients (14.3%) had major LEA and thirteen patients (6.4%) died during treatment (Figure 2A). Compared to 6.6% of major LEA and 3.5% in-hospital mortality in patients with grade 3 DFI (Figure 2A), patients with SIRS had significantly worse prognosis (*p* < 0.001). When individual components of SIRS criteria were studied, the presentation of abnormal temperature tended to have poor prognosis but did not reach statistical significance (*p* = 0.101) (Figure 2B).

**Figure 3:**
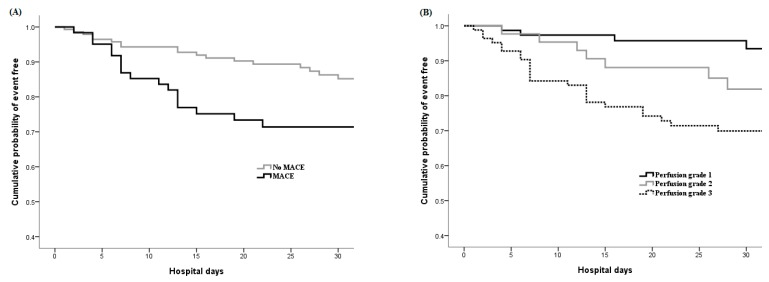
Kaplan-Meier curves of event free (major LEA or in-hospital mortality) in patients with SIRS stratified by (**A**) the association with MACE history and (**B**) the perfusion wound score.

The prognostic factors were analyzed between 42 and 161 patients with poor and better prognosis, respectively, in patients with SIRS (Table 2). The association with MACE history (52.4% versus 24.2%, respectively), retinopathy (73.8% versus 52.2%, respectively), poor peripheral circulation (grade 3 perfusion score 76.2% versus 39.1%, respectively), and with larger or deeper wounds were noted to have poor prognosis. Following multivariate logistic regression analysis, the grade 3 perfusion score (odds ratio 3.37, 95% confidence interval 1.04–10.94, *p* = 0.044), MACE history (OR 2.41, 95% CI 1.06–5.47, *p* = 0.036), and wound size (OR 1.003, 95% CI 1.00–1.01, *p* = 0.017) were the three independent risk factors predicting poor in-hospital prognosis in patients with SIRS (Table 2). Figure 3 demonstrates the 30-day accumulated event-free rate in these patients according to the association with MACE history (Figure 3A) and perfusion score (Figure 3B).

## 4. Discussion

The association with SIRS is not rare (28.1%) in patients with limb-threatening DFIs. This study further ascertains poor prognosis, both limb loss and in-hospital mortality for such patients, factors favoring SIRS presentations or poor prognosis have been reported.

This study found that age, wound size, and higher level of HbA1c at visiting were the factors favoring SIRS presentation. Subjects with SIRS were six years younger than those with grade 3 DFIs. The presentation of SIRS was association with the immune response and circulation cytokine [21] and younger subjects usually having stronger immune activity [22]. Inversely, aged patients with longer diabetes duration usually involved higher comorbidities and poor blood perfusion of foot. Both limited blood flow and less immune reaction in the wounds may regionalize the inflammatory response in elderly patients [23].

The large wound size and higher HbA1c level were also noted as factors prone to SIRS presentation. It is reasonable that more extensive tissue injury released more inflammatory mediators [24]. Though there is inconsistent association between HbA1c levels and LEAs prognosis reported by meta-analysis [25,26,27] and our observations [16,28], this study is the first to demonstrate higher HbA1c level has association with SIRS presentation in patients with limb-threatening DFIs. The HbA1c > 8.4% had valid predication of SIRS (sensitivity 67.7% and specificity 56.2%) among patients with DFIs. The higher quantities of cytokines, catecholamine, or cortisol secreted from inflammation stress of SIRS [29,30] may produce a secondary result in hyperglycemia. In addition, chronic hyperglycemia itself enhances cytokine and chemokine release, increased leukocyte marginalization, and exaggerated superoxide release [31], which might further exaggerate inflammatory responses in long-standing foot infections. The HbA1c itself has been reported as an acute phase protein that responds to inflammation conditions [32] independently of fasting blood glucose levels or obesity [25].

Since DFIs presenting SIRS has both limb- and life-threatening status, understanding the prognostic factors is important to improve treatments. This study identified perfusion grade 3, history of MACE and large wound size as independent risk factors for poor prognosis. Peripheral arterial disease itself has been reported to associate with high major LEA rate and high mortality [17,33,34]. Poor tissue oxygen tension might compromise the host local immune defenses and hinder the delivery of antibiotics to the infected tissue in patients with severe foot infections [23]. Patients with associated history of MACE might well be fragile and therefore have higher chance of PAD [35,36], thereby affecting the prognosis.

This study is limited by the single center and retrospective design. Nevertheless, to the best of our knowledge, this is the first large-series study to investigate severe infection of diabetic foot complications.

## 5. Conclusions

The presence of SIRS is a limb- and life-threatening condition for patients with DFI. Younger age, larger wound, and higher HbA1c levels at clinical visit favor the SIRS presentation. Meanwhile, association with MACE history or poor peripheral circulation foretells poor prognosis.

## Figures and Tables

**Table 1:** Clinical characteristics and wound classification between severe and grade 3 diabetic foot infection (DFI).

Characteristic	DFI Presenting SIRS (N = 203)		Grade 3 DFI (N = 519)		*p* Value
Age (years)	60.3	[52.5; 67.9]	66.0	[56.6; 76.3]	<0.001 *
Male gender	125	(61.6%)	319	(61.5%)	0.978
Diabetes duration (years)	12.0	[5.0; 20.0]	15.0	[7.0; 20.0]	0.040 *
Smoker	88	(43.3%)	204	(39.3%)	0.320
Hypertension	136	(67.0%)	367	(70.7%)	0.329
MACE ^a^ History	61	(30.0%)	187	(36.0%)	0.128
Retinopathy	115	(56.7%)	299	(57.6%)	0.815
Renal status					0.563
eGFR ≥60	84	(41.4%)	226	(43.5%)	
eGFR <60	84	(41.4%)	193	(37.2%)	
Dialysis	35	(17.2%)	100	(19.3%)	
HbA1c (%)	9.6	[7.6; 11.3]	8.1	[6.8; 10.16]	<0.001 *
Perfusion score					0.192
Grade 1	77	(37.9%)	187	(36.0%)	
Grade 2	31	(15.3%)	110	(21.2%)	
Grade 3	95	(46.8%)	222	(42.8%)	
Extent size (cm^2^)	15.0	[6.0; 45.0]	9.0	[4.0; 25.5]	<0.001 *
Depth/tissue loss score					0.175
Grade 1	9	(4.4%)	43	(8.3%)	
Grade 2	47	(23.2%)	124	(23.9%)	
Grade 3	147	(72.4%)	352	(67.8%)	
Sensation					0.156
Grade 1	100	(49.3%)	285	(55.1%)	
Grade 2	103	(50.7%)	232	(44.9%)	
Osteomyelitis	82	(40.4%)	202	(38.9%)	0.716
Days before visit	15.5	[9.0; 44.25]	23.5	[9.0; 60.0]	0.066
Hospital stay (days)	39.0	[24.0; 57.0]	26.0	[15.0; 40.0]	<0.001 *

^a^ Major adverse cardiac event including history of ischemic heart disease or coronary artery disease, cerebral vascular accident with embolic, ischemic, or hemorrhagic stroke; *, Significance: *p* value < 0.05. SIRS, systemic inflammatory response syndrome; DFI, Diabetic foot infection; MACE, major adverse cardiac events; and eGFR, estimated glomerular filtration rate.

**Table 2:** Factors analysis for prognosis in patients with SIRS.

Characteristic	Non-Amputation or Minor LEA ^a^ (n = 161)		Major LEA or Death ^b^ (n = 42)		*p* Value	Odds Ratio ^c^ (95% CI)	*p* Value
Age (years)	60.0	[50.3; 67.6]	62.2	[57.8; 73.3]	0.053		
MACE history	39	(24.2%)	22	(52.4%)	<0.001 *	2.41* (1.06–5.47)	0.036 *
Retinopathy	84	(52.2%)	31	(73.8%)	0.012 *	1.89 (0.81–4.41)	0.141
Renal status					<0.001 *		
eGFR ≥60	70	(43.5%)	14	(33.3%)		1	
eGFR <60	74	(46.0%)	10	(23.8%)		0.46 (0.17–1.24)	0.124
Dialysis	17	(10.6%)	18	(42.9%)		2.38 (0.88–6.47)	0.089
HbA1c (%)	9.7	[7.8; 11.4]	9.25	[7.23; 10.88]	0.220		
Perfusion score					<0.001 *		
Grade 1	72	(44.7%)	5	(11.9%)		1	
Grade 2	26	(16.1%)	5	(11.9%)		1.40 (0.32–6.24)	0.658
Grade 3	63	(39.1%)	32	(76.2%)		3.37* (1.04–10.94)	0.044 *
Extent size (cm^2^)	11.3	[4.98; 35.0]	44.5	[20.75; 102.38]	<0.001 *	1.003* (1.00–1.01)	0.017 *
Depth/tissue loss					0.038		
Grade 1	8	(5.0%)	1	(2.4%)		1	
Grade 2	43	(26.7%)	4	(9.5%)		0.95 (0.08–11.64)	0.989
Grade 3	110	(68.3%)	37	(88.1%)		1.93 (0.20–18.58)	0.568
Sensation					0.135		
Grade 1	75	(46.6%)	25	(59.5%)			
Grade 2	86	(53.4%)	17	(40.5%)			

* Significance: *p* value < 0.05; ^a^, 81 non-amputation and 80 minor-LEA; ^b^, 29 major-LEA and 13 death; ^c^, Association with poor treatment outcome (major LEA or death). Including continuous variables of extent size; and categorical variables of MACE history, retinopathy, renal function status, perfusion score, and depth/tissue loss.
